# Renal Leiomyosarcoma: A Diagnostic Challenge

**DOI:** 10.1155/2013/459282

**Published:** 2013-10-29

**Authors:** Jose R. Valery, Winston Tan, Cherise Cortese

**Affiliations:** ^1^Community Internal Medicine, Mayo Clinic, 4500 San Pablo Road, Jacksonville, FL 32224, USA; ^2^Hematology/Oncology, Mayo Clinic, 4500 San Pablo Road, Jacksonville, FL 32224, USA; ^3^Laboratory Medicine and Pathology, Mayo Clinic, 4500 San Pablo Road, Jacksonville, FL 32224, USA

## Abstract

Renal leiomyosarcoma is a very rare tumor that clinically and radiographically mimics more common renal malignancies. The infrequency of the condition makes it very difficult to diagnose. A 70-year-old male smoker presented with months of hematuria, right-sided flank pain, and weight loss. Imaging revealed a 3.8-centimeter renal mass that had characteristics similar to renal cell carcinoma. Initial biopsy of the mass was negative for malignancy. Two months later, subsequent imaging revealed what appeared to be metastatic bone lesions. Again, a biopsy of one of the lesions was negative for malignancy. Subsequent ureteral pyeloscopy, ureteroscopic renal pelvis biopsy, and brush cytology were negative for malignancy as well. The decision was made to perform nephrectomy for the removal of the mass. Pathologic analysis revealed renal leiomyosarcoma. This case illustrates the difficulty in diagnosing renal leiomyosarcoma. Repeated pathologic sampling was negative because of the tumor heterogeneity. Prompt diagnosis and treatment are very significant as surgical resection at an early stage offers the best prognosis.

## 1. Introduction

Leiomyosarcoma is a rare and aggressive smooth muscle cell tumor that can arise from different anatomic sites. They have a tendency to recur locally and metastasize early via the hematogenous route [1]. There are only a limited number of case reports and two case series on primary renal leiomyosarcoma. We present the case below to improve understanding of clinical presentation, radiographic findings, and pathologic features of this rare and aggressive tumor. 

## 2. Case Presentation

A 70-year-old Caucasian gentleman with a past history of smoking presented with a several-month history of right-sided flank pain, hematuria, and weight loss. 

Physical examination was unremarkable and did not reveal a palpable mass. His urinalysis was positive for occult blood. Renal profile, complete blood count, liver profile, and coagulation labs were all within normal limits at presentation. 

**Figure 1:**
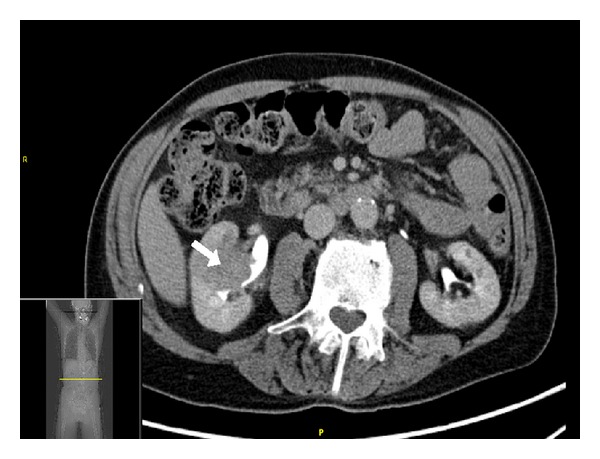
Computed tomography scan showing cross-section of the abdomen with a right-sided renal mass measuring 3.8 cm.

Imaging performed prior to presentation showed a 3.8-centimeter right-sided renal mass. At that point in time the differential diagnosis included renal cell carcinoma and invasive transitional cell carcinoma. However, biopsy of the mass was negative for malignancy showing smooth muscle bundles and benign parenchyma. Two months later, positron emission tomography-computed tomography (PET-CT) scan demonstrated a stable renal mass (Figure 1) and new PET-avid, bony lesions in the cervical and lumbar spine, ribs, and pelvis. CT-guided biopsy of a pelvic lesion was negative for malignancy. 

Suspicion for metastatic malignancy prompted further evaluation with ureteral pyeloscopy, ureteroscopic renal pelvis biopsy, and brush cytology. Brush cytology showed no evidence of malignancy and the renal pelvis biopsy showed urothelial cells without atypia. Despite the negative workup, it was not possible to rule out malignancy and the patient underwent a right-sided laparoscopic nephrectomy. 

**Figure 2:**
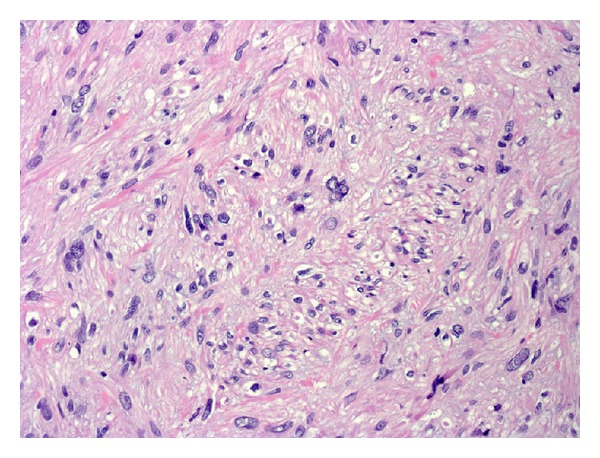
Spindled cells showing smooth muscle differentiation with cytologic atypia, H&E 20x.

**Figure 3:**
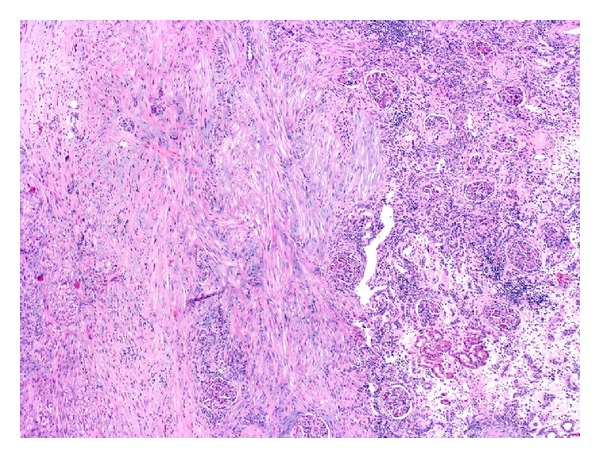
Invasion of leiomyosarcoma into the renal cortex, H&E 10x.

Pathological analysis of the specimen revealed a leiomyosarcoma (Figures 2 and 3), grade 2, measuring 4.0 cm, involving the hilum. There was moderate cytologic atypia with scattered mitotic figures and no areas of necrosis. Adjacent renal tissue showed invasion by the tumor. Actin and desmin immunohistochemistries were positive in tumor cells; CD117 and S-100 were negative. 

## 3. Discussion

Primary renal leiomyosarcoma presents a diagnostic challenge. It is a rare tumor with only 0.12% of renal malignancies confirmed as leiomyosarcoma [2]. There are no reliable clinical or radiologic features to distinguish leiomyosarcoma from more common renal malignancies such as clear cell carcinoma. As our case demonstrates, pathologic diagnosis prior to nephrectomy is difficult due to tumor heterogeneity leading to sampling difficulties. These tumors also have a variable degree of immunoreactivity for epithelial cell markers, further complicating pathologic analysis [2].

Clinical presentation is very similar to more common renal malignancies consisting of flank pain, hematuria, weight loss, and an abdominal mass. Typically occurring between the fourth and eighth decades of life, renal leiomyosarcoma is more common in women and arising from the right kidney [3]. It can sometimes present as spontaneous rupture of the kidney [4]. 

Computed tomography imaging of renal sarcomas usually reveals a solid infiltrating mass which is often very difficult to distinguish from lymphomas. Findings consistent with fat or bone on imaging may suggest liposarcoma or osteosarcoma. However, radiographic features are typically indistinguishable from renal cell carcinoma. Although the study of choice, computed tomography is not able to distinguish renal LMS from more common malignancies [1]. 

Accurate diagnosis has very significant implications for prognosis and treatment. Renal leiomyosarcoma has a grim prognosis with a median survival between 17.9 and 25 months [3]. Treatment of leiomyosarcoma consists of radical nephrectomy. Surgery followed by chemotherapy and radiation has been advocated in the past with limited success [3, 5]. Radical operations, size <5 cm, low histologic grade, and negative lymph node metastasis are all associated with a better prognosis [5].

## 4. Conclusion

Our case demonstrates the difficulty in making a tissue diagnosis of primary renal leiomyosarcoma prior to nephrectomy. This illustrates the point that several biopsies may not be helpful. In cases where clinical suspicion for malignancy is high, nephrectomy should be performed in a timely manner.

## Clinical Practice Points

- Primary renal leiomyosarcoma (LMS) is a rare tumor.
- LMS is a challenging diagnosis to make prior to tissue biopsy. This is due to the fact that clinical presentation and initial imaging are usually not helpful in distinguishing LMS form more common renal malignancies.
- Biopsy is needed for diagnosis. may require nephrectomy for diagnosis as false negative biopsies occur frequently (as in our case).
- Surgery is the main treatment modality for renal LMS.
